# Towards multifunctional inorganic materials: biopolymeric templates

**DOI:** 10.3762/bjnano.6.172

**Published:** 2015-08-05

**Authors:** Claudia Steinem, Joachim Bill

**Affiliations:** 1 Institute of Organic and Biomolecular Chemistry, Georg-August University Göttingen, Tammannstr. 2, 37077 Göttingen, Germany; 2 University of Stuttgart, Institute of Materials Science, Heisenbergstr. 3, 70569 Stuttgart, Germany

Inorganic functional materials are widely applied due to their electrical, optical, magnetic and mechanical properties. Accordingly, these materials exhibit an enormous impact on key technologies relevant for future fields such as energy generation and storage, information and medical technology as well as automotive engineering. The manufacturing of such materials is usually performed at elevated temperature and/or pressure combined with enormous experimental effort and extensive equipment. Consequently, problems arise due to shrinkage or grain growth. In addition, the production of composite materials with multifunctional properties may be severely limited because of the mismatch between the thermal expansion coefficients of the different components. This is also an issue in the combination of inorganic components with temperature sensitive materials, which is limited or even impossible.

In contrast to the aforementioned manufacturing methods, biomineralization leads to fascinating, complex-structured, inorganic materials under ambient conditions. The corresponding processes have been evolutionarily optimized over millions of years and involve biopolymeric templates, which control the mineralization and the structure formation of inorganic components in an aqueous environment. Accordingly, composites made of inorganic solids (i.e., calcium phosphate or carbonate) and biopolymers are formed. Furthermore, the resulting combination of inorganic and bioorganic components yields biominerals with unique multifunctional features and an expanded spectrum of properties as compared to those of the pure inorganic components.

Although the composition of biominerals comprises only a limited number of chemical elements, the principles of biomineralization and the obtained structures can be considered archetypes for the generation of inorganic functional materials not found in living nature. This highly topical research field is currently drawing worldwide attention and is a main subject of the Priority Program 1569, “Generation of multifunctional inorganic materials by molecular bionics”, of the Deutsche Forschungsgemeinschaft in Germany. Correspondingly, this Thematic Series addresses multifunctional, inorganic materials generated by templating with biomolecules. The reader of this series will gain a comprehensive overview about the general ideas and principles of biopolymeric templating by means of selected examples presented in the contributions.

We would like to acknowledge all contributors for their invaluable participation in this Thematic Series and thank the referees for their great support to ensure that this Thematic Series fulfills high quality standards. Many thanks also go to the Beilstein Journal of Nanotechnology for providing us with the opportunity to present this novel, exciting research field to a broad audience and in particular Dr. Uli Fechner from the Editorial Team for his guidance and support.

Claudia Steinem and Joachim Bill

Göttingen and Stuttgart, June 2015

